# Integrated monitoring of AMR and enterotoxins genes of S. aureus isolated in Lombardy

**DOI:** 10.1093/eurpub/ckac131.408

**Published:** 2022-10-25

**Authors:** E Spinelli, G Magagna, M Tilola, F Rossi, V Filipello, F Guarneri, N Formenti, A Ianieri, GL Alborali, G Finazzi

**Affiliations:** 1 Food Safety, IZSLER, Brescia, Italy; 2 CRESA, Milan, Italy; 3 Territorial Site of Brescia, IZSLER, Brescia, Italy; 4 UNIPR, University of Parma, Parma, Italy

## Abstract

**Background:**

S. aureus is a widespread pathogen responsible for mild to severe human and animals’ infections. The abuse of antimicrobials provides the potential for selection of resistant strains in livestock, which represents a public health concern. S. aureus can carry several virulence factors of which staphylococcal enterotoxins (SEs) play a key role during food poisoning in human populations. The aim of this study is to monitor the prevalence of antimicrobial resistance factors in S. aureus isolates and their ability to produce enterotoxins.

**Methods:**

Within an ongoing monitoring plan for the assessment of antimicrobial resistance in S. aureus strains, a total of 83 isolates collected from food, and swine and dairy farms, between 2020-2022, were characterized using MLST and then screened for the presence of methicillin resistance and SEs genes. The isolates were tested for susceptibility to a panel of 14 antimicrobial agents using the disc agar diffusion method on Mueller-Hinton agar.

**Results:**

Among 83 S. aureus isolates, 53% carried at least one SEs gene. Eighteen isolates were methicillin-resistant of which 17 were no-enterotoxigenic strains belonging to ST398, and one was a food origin ST8 strain and harbored SEs genes. Among the ST398 isolates, only one was a food origin strain, while the others were from swine farms. The antibiogram showed that a few isolates were susceptible to nalidixic acid, and 42% resulted multidrug-resistant.

**Conclusions:**

Our results showed that more than half of S. aureus isolates were enterotoxigenic, the majority belonging to food industries. Numerous tested isolates resulted multidrug-resistant, confirming that antimicrobial resistance is a critical public health threat in a food safety perspective.

**Key messages:**

• The antimicrobial resistance profiles of S. aureus isolates underlines the importance of monitoring plans with a One Health perspective.

• The prevalence of enterotoxins genes in S. aureus strains in Lombardy confirms the relevance of the microorganism as a foodborne pathogen.

